# A modification of the constant-head permeameter to measure saturated hydraulic conductivity of highly permeable media

**DOI:** 10.1016/j.mex.2017.02.002

**Published:** 2017-02-14

**Authors:** Jelmer J. Nijp, Klaas Metselaar, Juul Limpens, Harm P.A. Gooren, Sjoerd E.A.T.M. van der Zee

**Affiliations:** a Wageningen University, Soil Physics and Land Management Group, Wageningen, The Netherlands; b Wageningen University, Plant Ecology and Nature Conservation Group, Wageningen, The Netherlands; c Wageningen University, Soil Geography and Landscape Group, Wageningen, The Netherlands; d Monash University, School of Chemistry, Melbourne, Victoria 3800, Australia

**Keywords:** Low-resistance permeameter, Permeability, Low-resistance permeameter, Laboratory method, Soil physics, Reynolds number, Compression, Peat, *Sphagnum*

## Abstract

The saturated hydraulic conductivity (*K_s_*) is a key characteristic of porous media, describing the rate of water flow through saturated porous media. It is an indispensable parameter in a broad range of simulation models that quantify saturated and/or unsaturated water flow.

The constant-head permeameter test is a common laboratory method to determine K_s_ on undisturbed soil samples collected from the field. In this paper we show that the application of this conventional method may result in a biased *K_s_* in the case of highly permeable media, such as the top layer of *Sphagnum* peat and gravel. Tubes in the conventional permeameter, that collect water under the sample, introduce a hydraulic head-dependent resistance for highly permeable media and result in an underestimation of *K_s_*.

We present a simple and low-budget alternative of the constant-head permeameter test that overcomes the disadvantages of conventional permeameters. The new method was successfully tested on intact highly permeable peatmoss collected from a northern peatland.

• Conventional constant-head permeameters underestimate *K_s_* of highly permeable media due to flow resistance in tubing systems
• We developed the low-resistance permeameter to overcome this disadvantage.
• Testing of the low-resistance permeameter demonstrated no systematic bias and successful application for highly permeable media.

## Graphical abstract

**Figure:**
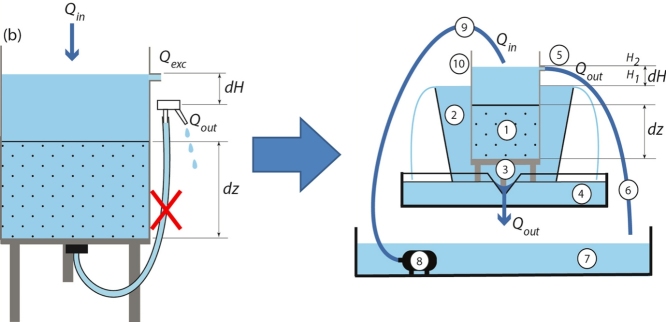

## 1 Method details

### 1.1 Conventional constant-head permeameter

The constant-head permeameter is a broadly used laboratory instrument to determine the saturated hydraulic conductivity (K_s_) in laboratory conditions. The procedure is well-documented [1], [2], [3] and standardized (e.g. ISO/TS 17892-11 [4], ISO/FDIS 17312 [5], and NEN5789 [6]).

The hydraulic conductivity can be determined using Darcy’s law (Eq. (1)) and measured data of the water flow rate (*Q_out_*; L^3^T^−1^) through a sample, the sample cross section area (*A*; L^2^) the difference in hydraulic head (*dH*; L) and the distance over which *dH* is applied (*dz*; L).

(1) $$Q_{out}=-AK_{s}\frac{dH}{dz}$$

**Fig. 1:**
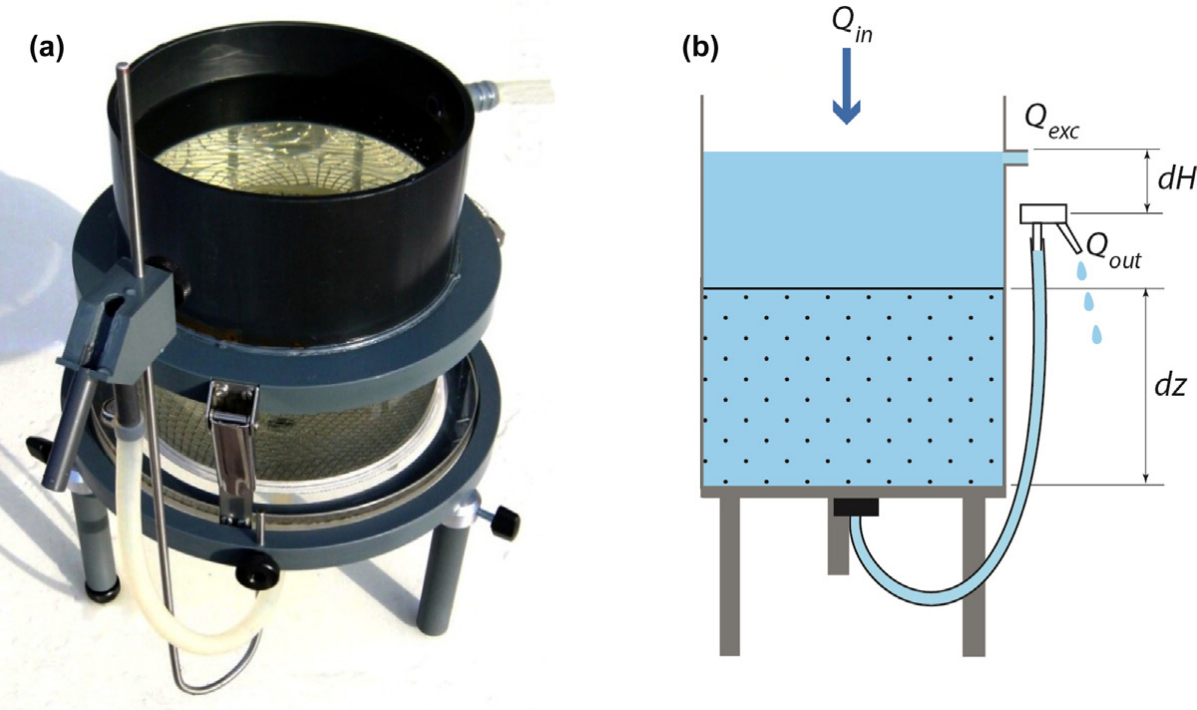
Conventional constant-head permeameter equipment (a; modified with permission from Wageningen Environmental Research (Alterra); [7]) and schematic representation showing the tubes collecting the outflow water (b).

To that end, an undisturbed soil sample is placed in a PVC sample ring (Fig. 1). To establish a pressure head difference, another PVC ring is clamped watertight on top of the sample ring and filled with water (*Q_in_*). Water is caused to flow through the sample and collected at the downflow end of the sample ring through a tubing system to an outlet. The rate of water flow *Q_out_* can be adjusted by modifying the pressure head difference *dH*, which can be achieved by adjusting the height of the tube outlet. The hydraulic head difference is kept constant through time, by a continuous supply of water from above in combination with an overflow outlet (*Q_exc_*). In a closed system the excess water can be collected and repumped on top of the water column. In the conventional constant-head permeameter tests it is assumed that *K_s_* is independent on all other parameters, such as the hydraulic head difference, and that the sample is the only resistance to water flow. To obtain an accurate *K_s_* estimate, the applicability of the constant-head permeameter is constrained to hydraulic head differences larger than 0.01 m (i.e. hydraulic gradients larger than 0.1 m m^−1^). Smaller hydraulic head differences cannot be accurately estimated and introduce considerable error.

## 2 Application of the constant-head permeameter on highly permeable media: living *Sphagnum* peat

An example where adequate estimation of hydraulic conductivity plays a central role is in the hydrology of northern peatlands [8], [9], [10]. Northern peatlands fix large quantities of carbon and represent a key component of the global carbon cycle [11], [12]. Carbon is stored through the process of photosynthesis, during which CO_2_ is transferred into organic matter, which will be (partially) decomposed and accumulate as peat. Photosynthesis, hence CO_2_ uptake, predominantly takes place by peatmosses, which represent the dominant vegetation in northern peatlands. During the last 10,000 years, northern peatlands acted as a net carbon sink. Besides light availability, wet surface conditions and frequent rain are crucial factors affecting peatmoss growth and to sustain net carbon uptake [13], [14], [15]. However, climate change is projected to result in increased temperatures and more frequent droughts [16], and thereby puts the carbon uptake of northern peatlands under pressure [17], [12]. Hydrological models are required to predict how climate change affects the water balance of northern peatlands, in which appropriate parameterization of the saturated hydraulic conductivity of the highly permeable (porosity over 0.80; e.g. Kellner [18] and Price & Whittington [19], unhumified (i.e. undecomposed), living peatmoss layer plays an essential role [9].

**Fig. 2:**
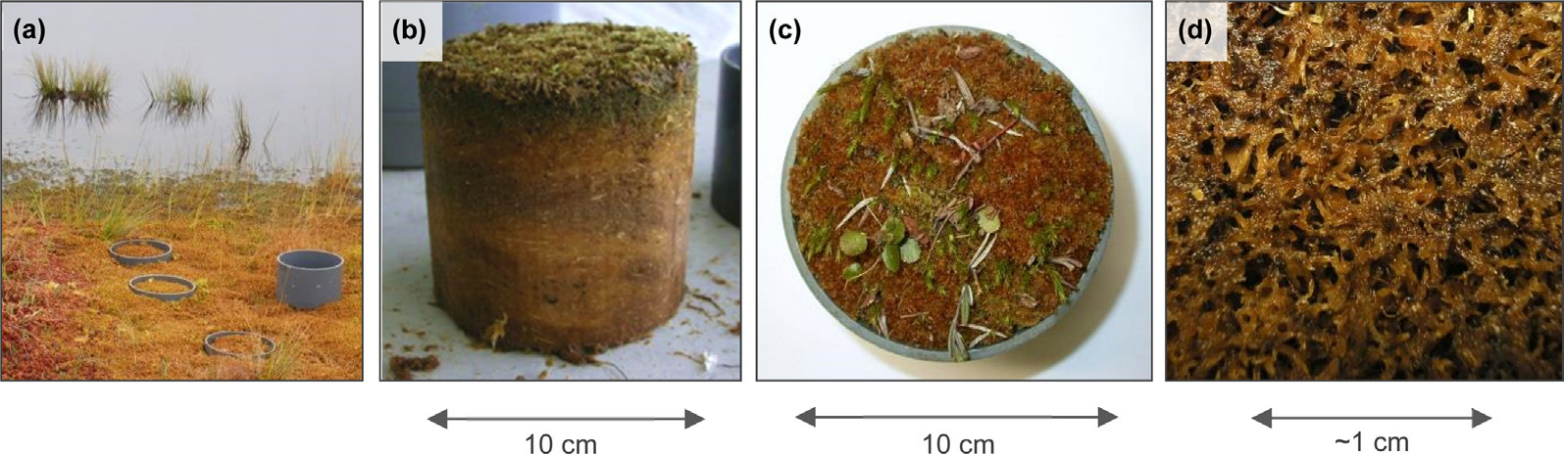
Peatmoss in field conditions (a), frozen peatmoss subsample (b), topview on sample placed in PVC ring (c) and example of the porous peatmoss structure (d).

To quantify the hydraulic conductivity of the highly porous living peatmoss layer, we collected undisturbed samples (diameter 0.2 m) of the top 0–0.1 m of *Sphagnum* peat (i.e. the living, unhumified, peatmoss layer) from Degerö Stormyr (64°N 19°E), an undisturbed oligotrophic minerogenic peatland in northern Sweden. PVC rings were gently pushed in the peat while cutting the peat around the PVC edges with a serrated knife. Samples were frozen and transported with a cooling van to the Soil Physics Laboratory in Wageningen, The Netherlands, to prevent disturbance of the pore structure. To minimize the effect of sampling induced disturbance, a subsample (diameter 0.1 m) was taken of the frozen peatmoss by using a band saw (Fig. 2). A filter cloth at the bottom prevented the peat material from falling out of the sample ring. After defrosting, samples were slowly saturated from below to prevent entrapment of gas bubbles, which may reduce *K_s_* by a factor of five to about forty [20], [21], [22].

### 2.1 Complications when applying constant-head permeameter on peatmoss

**Fig. 3:**
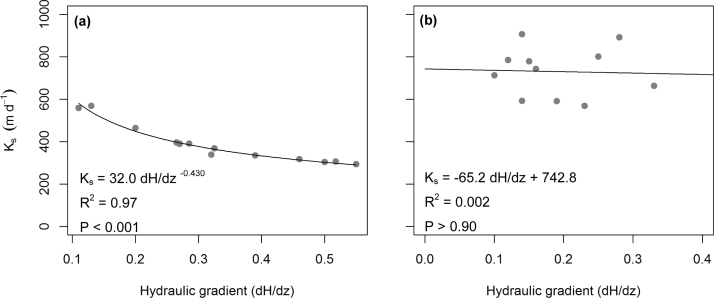
Relation between hydraulic gradient and saturated hydraulic conductivity when adopting the conventional constant-head permeameter (a) and the newly developed low-resistance permeameter (b).

Several initial constant-head permeameter tests were done on one sample but with varying hydraulic gradient (0.1–0.6 m m^−1^) to test adequate functioning of the permeameter. During those tests, we found that *K_s_* depended on the applied pressure head difference (Fig. 3a) instead of being an intrinsic material constant, as it is supposed to be. The established relation was non-linear, with larger hydraulic head differences resulting in lower *K_s_*. Below we discuss the mechanisms that may cause the observed non-linearity.

#### 2.1.1 The effect of compression

Peat soils are known for their large compressibility [23], [9], [24]. Following classic soil mechanics [25], the bulk density (and hence available pore space) of peat will decrease with increases in effective stress (*σ*′). The effective stress is a function of the total stress (i.e. external loads; *σ*) and the pore water pressure (*h*) acting upon the peat matrix (Eq. (2)).

(2) $$\sigma^{\prime }=\sigma -h$$

With increased effective stress the peat matrix compresses, resulting in a decrease in hydraulic conductivity [24]. Moreover, field measurements indicate that the estimated K_s_ depends on the imposed hydraulic head, which was attributed to the large compressibility of peat [26], [27], [28].

Also with constant-head permeameter tests a larger hydraulic head difference will reduce the pore water pressure in the sample, increase the effective stress, and compress the peat matrix. It is uncertain, however, to what extend (very) small differences in imposed hydraulic head in laboratory conditions affect the degree of compaction and associated reduction in *K_s_*.

#### 2.1.2 Non-Darcian flow?

Darcy’s law is based on the assumption of laminar flow. In highly permeable materials, large hydraulic gradients (i.e. high flow rates) may result in non-laminar (i.e. turbulent) flow, resulting in energy dissipating by internal frictional forces of water, or interfacial drag-forces [29] which in turn reduces *K_s_*. To test whether the non-linear behaviour in Fig. 3a may have resulted from non-laminar flow, we calculated the Reynolds number (Eq. (3))

(3) $$Re=du\frac{\rho }{\eta }$$

where *d* is the effective pore diameter (m), *u* the specific discharge (m s^‐1^), *ρ* the density of water (kg m^−3^) and *η* the dynamic viscosity (N s m^‐2^). For porous media, it is generally accepted that turbulent flow may occur if *Re* > 1 [30]. The effective pore diameter was estimated from water retention curves for peatmoss using the Young-Laplace equation of capillarity (See Appendix 1 for calculation details in Supplementary material). The Reynolds number for the *Sphagnum* peat was 0.025–0.65, suggesting that the assumption of laminar flow holds and the application of Darcy’s law is valid.

#### 2.1.3 Collection tubes introducing extra flow resistance

The observation that the hydraulic conductivity decreases with increasing hydraulic gradient matches well with Bernoulli’s principle, which states that an increase in pressure results in a decrease of fluid speed [32]. As the cross sectional area of the tubes collecting outflow from the sample is about 400 times smaller than the cross sectional sample area, large volumes of water need to be transported through the tubes and flow velocity is high. The Reynolds number for flow through the collection tubes is well above 4000 (Eq. (2)), indicating that water flow in tubes was turbulent and the tube walls represent a resistance to flow. The derivation of *K_s_* is based on the sum of all resistances in the permeameter, including those of the collection tubes. In case of highly permeable media, with high flow rates, the importance of the resistance in such tubing system increases non-linearly with larger hydraulic gradients differences, resulting in an increasingly larger underestimation of *K_s_*.

## 3 Development of the low-resistance permeameter

### 3.1 General description

An alternative to overcome the bias introduced by tubing systems is to use the open outflow permeameter, which functions the same as the constant-head permeameter, but avoids tubing systems as it has free drainage at the bottom of the sample (see e.g. [1]; pp 78–79). With the open outflow permeameter, however, only large hydraulic gradients can be applied. Highly permeable media, such as gravel and the topsoil of *Sphagnum* peat, may exhibit non-Darcian flow at large hydraulic gradients [30]. In practice, however, hydraulic gradients are very small in natural systems, especially in the flat landscapes of northern peatlands [33]. Using the open outflow permeameter may thus result in unnatural non-Darcian behaviour for highly permeable media.

**Fig. 4:**
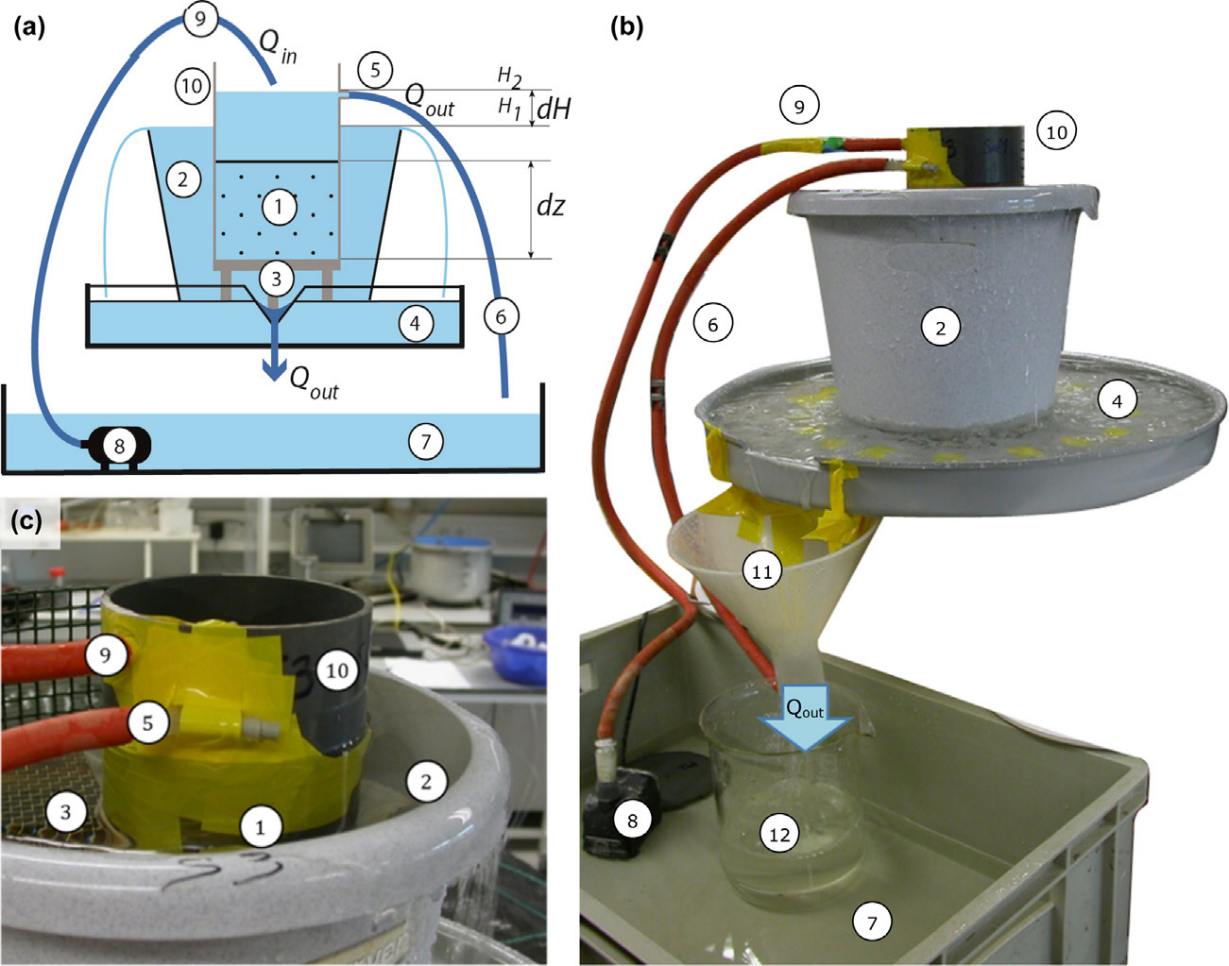
Schematic (a), representational (b) and zoomed (c), overview of the low-resistance permeameter. 1: soil sample in PVC ring, 2: sample reservoir, 3: grid on height-adjustable tripod, 4: collection reservoir, 5: overflow tube to maintain a fixed hydraulic head difference, 6: tube to discharge overflow water, 7: overflow reservoir, 8: water pump, 9: inflow tube, 10: top PVC ring used to establish water column on top of sample, 11: Funnel collecting outflow water, 12: Beaker to measure outflow volume.

A modified permeameter is thus required that has 1) no tubes nor other large resistances in the flow system and 2) the possibility to apply small hydraulic gradients. We therefore designed the ‘low-resistance permeameter’: a simple and low-cost adaptation of the existing constant-head permeameter (See Fig. 4). By fulfilling the two criteria above, the low-resistance permeameter combines the two strengths of the constant-head permeameter and open-outflow permeameter.

### 3.2 Removal of resistances to flow

After sample preparation following the protocol described in Section 2, the sample is placed on a grid platform in a reservoir (referred to as sample reservoir) (Fig. 4). The sample reservoir was filled with water until water starts to flow over the rim. Next, the water flowing over the rim was collected by another reservoir with a larger diameter (collection reservoir 1) under the sample reservoir. A wide notch in collection reservoir 1 directed the water into an Erlenmeyer flask to measure outflow, introducing as little flow resistance as possible. In this way, the need for collection tubes and hence additional flow resistance is avoided (assuming resistance of the sample reservoir rim to water flow is negligible as compared to that of the collection tubes and filter cloth). If no Erlenmeyer flask is placed under the outflow funnel, water flowed to the overflow reservoir, from which water could be recycled to the top of the sample.

### 3.3 Application of small hydraulic gradients

A small difference in hydraulic head is established as follows: On top of the sample ring, another sample ring with the same diameter is connected water-tight. Similar to the constant-head permeameter, this ring is used to apply a hydraulic head difference by establishing a water column on top of the sample. The first hydraulic head (H_1_) is defined by the rim of the sample reservoir (Fig. 4). The water inflow is regulated by a pump so that water enters on top of the sample. The water level rises until it reaches the outlet. In this way a constant water level is created on top of the sample. This provides the second value of the hydraulic head to be used in the calculations (H_2_). The hydraulic gradient can be adjusted by modifying the height of the grid platform on which the sample is placed. It should be noted, however, that it is recommended to not employ too small hydraulic gradients, as the relative importance of measurement errors increases. With a hydraulic head difference of 0.02 m over a sample of 0.10 m (i.e. a hydraulic gradient of 0.2), a measurement error of 0.001 m results in a 5% over- or underestimation of *K_s_*.

### 3.4 Measurement preparation

1 Connect the top ring to the sample ring water-tightly.
2 Put a filter cloth (mesh size ≈ 0.001 m) beneath the sample with an elastic around the sample ring to prevent the sample from falling out of the sample ring.
3 Place the sample on the grid platform (non-ferrous or plastic; mesh size >0.005 m). Make sure the grid is horizontally levelled.
4 Add water (representing the chemical composition under field conditions to prevent pore dilation [34]) and raise the water level till the bottom of the sample. Slowly saturate the sample from below to prevent air entrapment. In highly permeable soils, saturation is relatively quick as compared to fine grained soils.
5 Fill the collection reservoir and add sufficient water to the overflow reservoir that can be pumped and recycled to the top of the sample.
6 Connect the tube (6) to the water inlet and let water enter on top of the sample. Make sure no water enters directly on top of the sample but rather via the wall of the top PVC ring to avoid disturbing the soil structure. Start with low pumping rates until a water layer establishes.

### 3.5 Measurement procedure

1 Measure the sample height, i.e. the distance over which the pressure difference is being applied (*dz*) and the sample area (*A*).
2 Measure the distance between the water level **in** the top PVC ring till the rim of the top PVC ring (for *H_2_*). Also measure the distance between the water level **outside** the top PVC ring till the top of the PVC ring (for *H_1_*). The hydraulic head difference is represented by *H_1_* − *H_2_*.
3 Put a beaker or Erlenmeyer flask (volume of about 2 l) under the outlet of the collection reservoir to measure outflow and record the time between placement and removal. Similar to other measurement protocols, the hydraulic conductivity can be reliably estimated once 3 subsequent outflow measurements deviate less than 10% from each other by taking the logarithmic average.

## 4 Validation of the low-resistance permeameter

Using another intact *Sphagnum* peat sample, we tested the overall performance of the low-resistance permeameter and whether the non-linear effect of hydraulic gradient on *K_s_* persisted. In the range of the employed hydraulic gradients of 0.1–0.4 (i.e. a hydraulic head difference of 1–4 cm) we found no significant relation between *dH/dz* and *K_s_* (Fig 3b; Linear regression; slope P = 0.90).

Still, quite some variation around the mean *K_s_* of 730 m d^‐1^ was observed. The 95% confidence interval is 69 m d^−1^, which deviates less than 10% from the mean and is acceptable. Variation could be caused by a slow decline in *K_s_* over multiple days due to collapse of pores or pore dilation [34]. Additionally, recirculation of water without removal of (eroded) peat particles may result in clogging of pores, leading to a decrease in *K_s_* over time. This test of principle is based on a single and different peatmoss core for each of the two methods, and different samples would yield different values of *K_s_* due to spatial heterogeneity. Nevertheless, the results clearly demonstrate that the adjustments incorporated in the newly developed low-resistance permeameter prevent systematic underestimation of *K_s_*. This also indicates that, within the pressure range applied (Fig. 3), the large compressibility of peat is not the key factor explaining the dependence of *K_s_*.

## 5 Concluding remarks

For many permeable media, the constant-head permeameter is a suitable method to determine the saturated hydraulic conductivity (*K_s_*). However, for highly permeable soils, resistances to flow in tubing systems of conventional constant-head permeameters may result in an underestimation of *K_s_*. The underestimation becomes non-linearly more severe with larger hydraulic gradients. Highly permeable soils occur in a broad range of conditions, ranging from gravel and green roof substrates, to the topsoil of ombrotrophic peatlands dominated by peatmoss.

Our newly developed method suggests that care should be taken when interpreting literature values of *K_s_* for highly permeable soils. Such literature values may be too low if *K_s_* is estimated using the conventional constant-head permeameter, due to additional resistances in the flow system. For the accurate estimation of *K_s_* for highly permeable soils it is highly recommended to employ the presented low-resistance permeameter modifications presented here. This recommended modification has implications for a broad range of disciplines in which the saturated hydraulic conductivity plays an essential role.
