# Supplementary material for: A modification of the constant-head permeameter to measure saturated hydraulic conductivity of highly permeable media
Source: MethodsX. 2017 Feb 14;4:134–42. doi: 10.1016/j.mex.2017.02.002 (PMC5345952; doi:10.1016/j.mex.2017.02.002)
Supplement: Supplementary file 1 [file mmc1.docx]

**Appendix 1: Calculating the Reynolds numbers for permeable media**

To determine whether turbulent flow occurred in the tubing system of the conventional permeameter or in the peatmoss sample in the low-resistance permeameter, the Reynolds number ($Re$) was calculated following Hillel (2004) as

$$Re=\frac{2r u \rho}{\eta} (Eq. 1)$$

where $r$ is the effective pore radius (m), $u$ the mean flow velocity in the pores (m s^-1^), $\rho$ the density of water (kg m^-3^) and $\eta$ the dynamic viscosity (0.001002 N s m^-2^ at 20 °C). Reynolds number values < 1 indicate that flow in soil pores is laminar (Hillel, 2004), and that Darcy’s law can be applied to estimate the saturated hydraulic conductivity. The effective pore diameter was derived from the water retention curve of the living peatmoss layer (see Nijp *et al.* (2016) for details) as follows. The water content of a sample at given matric potential $h$ (m) is a function of the sizes and volumes of the water-filled pores, which can be described with the Young-Laplace equation of capillarity (Eq. 2).

$$h=\frac{2\sigma cos\varphi}{\rho gr} (Eq. 2)$$

Here, $\sigma$ the surface tension (J m^‑2^), $\varphi$ the contact angle (in degrees), $g$ the gravitational constant (9.81 m s^-2^) and $r$ the radius of the capillary tube (m). The contact angle ranges from 0° for living peatmoss (Valat *et al.*, 1991) to 73 – 88° for peat (Thompson & Waddington, 2008; Waniek E. *et al.*, 2000). The effective pore radius of the peat sample ($r_{p}$)was defined as the pore radius at which the sample contains 50% of the total water content. Assuming pores were continuous and parallel, $r_{p}$ could be estimated from the cumulative pore size distribution function. For calculating $Re$ for the tubing system, $r$ represents the tube radius ($r_{t}$).

The mean flow velocity $u$ for the tubing system in the conventional constant-head permeameter was calculated by dividing the measured outflow $Q$ (m^3^ s^-1^) by the surface area $A$of the tubes. For the peat sample, the mean flow velocity in individual pores was calculated by dividing the mean flow velocity of the peat sample ($Q/A$) over the number of pores. In turn, still under the assumption that pores were continuous and parallel, the number of pores was estimated by dividing the porous surface area of the sample (sample area $A$ (m^2^) multiplied by porosity $\phi$) over the estimated mean pore surface area (Eq. 3).

$$\frac{\phi A}{{{\pi r}_{p}}^{2}} (Eq. 3)$$

Based on the acquired data, the approach outlined above, $Re$ varied between 0.025 and 0.65 for water flow in individual pores in the peat sample, dependent on the contact angle used. This indicates that the assumption of laminar flow holds and Darcy’s law can be applied to estimate the saturated hydraulic conductivity. Conversely, the average $Re$ in the tubing system was 1.5·10^4^, which well exceeds the turbulent flow regime threshold $Re>4000$ for cylindrical channels (Czachor, 2011). Hence, flow in the tubing systems is turbulent, implying that the rate of flow is not proportional to the hydraulic gradient. As a consequence, constant-head permeameters with tubing systems collecting outflow from the sample will yield an underestimated saturated hydraulic conductivity of highly permeable porous media.

**References**

Czachor H (2011) Laminar and Turbulent Flow in Soils. In: *Encyclopedia of Agrophysics*, pp 413-413, Springer Netherlands, Dordrecht.

Hillel D (2004) *Introduction to environmental soil physics*, Elsevier Academic Press, Amsterdam, NL.

Nijp JJ, Metselaar K, Limpens J *et al.* (2016) Including hydrological self-regulating processes in peatland models: effects on peatmoss drought projections. *Science of The Total Environment,* **580**, 1389–1400.

Thompson DK, Waddington JM (2008) Sphagnum under pressure: towards an ecohydrological approach to examining Sphagnum productivity. *Ecohydrology,* **1**, 299-308.

Valat B, Jouany C, Riviere LM (1991) Determination of the wetting properties of air-dried peats and composts. *Soil Science,* **152**, 100-107.

Waniek E., Szytalowicz J., T. B (2000) Determination of soil-water contact angle in peat-moorish soils by capillary rise experiments. *Suoseura,* **51**, 149–154.
